# On the Treatment of Teeth When the Nerve Is Endangered by Caries

**Published:** 1856-04

**Authors:** 


### ARTICLE VI.

### *On the Treatment of Teeth when the Nerve is endangered by Caries.*

Believing the vitality of the pulp of a tooth to be absolutely important to its highest state of health, as well as its greatest permanence, I have always endeavored to preserve that vitality when possible.

Various expedients were resorted to in my earlier practice to effect this object, until I adopted a method of treatment, which, up to the present time, has constantly given me reason to believe it the most generally effective of any method known, at least to myself. It consists only in taking advantage of the protecting and recuperative power of nature, which she is so ready to exert when permitted.

It was while examining, with curiosity, this power, in relation to the formation of secondary dentine in teeth where the nerve pulp had been endangered but not morbidly affected, that the idea occurred to me of turning it to some practical purpose.

I prejudged, that, in cases where the nerve being very nearly exposed by the advance of decay, if the diseased action could be retarded, the pulp would improve the time so gained to throw out its defence against the approaching danger, in the form of secondary dentine.

With this idea I commenced the trial. At first, after removing as much of the caries as could be done without injuring the nerve, I made use of various applications having a tendency to arrest or retard decay; but later, adopted astringents and chlorine solutions mainly; which I still continue with a success that often surprises me.

The cavity having been thus prepared and treated, is made dry and stopped* temporarily, for from two to six months, the time depending on the apparent activity of disease, when the stopping is removed, further excavation made if it is found safe to do so, the applications renewed, and the cavity again stopped.

This process is repeated until the caries ceases to be active in its character, and a solid floor of dentine is found in the cavity. Then thorough excavation is performed and a permanent gold filling put in. The tooth thus retains its full natural health and permanency, as if the nerve had never been endangered.

I find the most convenient material for a temporary stopping, to be gutta percha, with sufficient fine spar or silex mixed with it to remove its elasticity when warmed. A portion of oxyd of tin, mixed with the same, renders it perhaps more preservative, but may be objectionable from its liability to produce discoloration.

***I use the word stopping, for the temporary, and filling, for the permanent operation.**

